# A Transdiagnostic group therapy for sleep and anxiety among adults with substance use disorders: Protocol and pilot investigation

**DOI:** 10.3389/fpsyt.2023.1160001

**Published:** 2023-03-29

**Authors:** Melissa E. Milanak, Sara M. Witcraft, Jie Young Park, Katharine Hassell, Tierney McMahon, Allison K. Wilkerson

**Affiliations:** ^1^Department of Psychiatry and Behavioral Sciences, Medical University of South Carolina, Charleston, SC, United States; ^2^Edward Via College of Osteopathic Medicine–Carolinas, Spartanburg, SC, United States; ^3^Lutheran Homes of South Carolina, Mount Pleasant, SC, United States; ^4^Department of Psychology, Northwestern University, Evanston, IL, United States

**Keywords:** substance use disorder, anxiety, insomnia, transdiagnostic, treatment development

## Abstract

**Introduction:**

Treatment of substance use disorders (SUDs) is challenging with high rates of treatment dropout and relapse, particularly among individuals with comorbid psychiatric conditions. Anxiety and insomnia are prevalent among those with SUD and exacerbate poor treatment outcomes. Interventions that concurrently target anxiety and insomnia during the early stages of SUD treatment are lacking. To this end, we investigated the feasibility and preliminary effectiveness in a single-arm pilot trial of an empirically informed group transdiagnostic intervention, Transdiagnostic SUD Therapy, to concurrently reduce anxiety and improve sleep among adults receiving treatment for SUD. Specifically, we hypothesized that participants would evidence declines in anxiety and insomnia and improvements in sleep health, a holistic, multidimensional pattern of sleep-wakefulness that promotes wellbeing. A secondary aim was to describe the protocol for Transdiagnostic SUD Therapy and how it may be implemented into a real-world addiction treatment setting.

**Method:**

Participants were 163 adults (*M_age_* = 43.23; 95.1% White; 39.93% female) participating in an intensive outpatient program for SUD who attended at least three of four Transdiagnostic SUD Therapy sessions. Participants had diverse SUDs (58.3% alcohol use disorder, 19.0% opioid use disorder) and nearly a third of the sample met criteria for two SUDs and comorbid mental health diagnoses (28.9% anxiety disorder, 24.6% major depressive disorder).

**Results:**

As anticipated, anxiety and insomnia reduced significantly across the 4-week intervention period from clinical to subclinical severity, and sleep health significantly improved (*p*s < 0.001). These statistically significant improvements following Transdiagnostic SUD Therapy demonstrated medium to large effects (*d*s > 0.5).

**Conclusion:**

Transdiagnostic SUD Therapy is designed to be flexibly administered in “real-world” clinical settings and, preliminarily, appears to be effective in improving emotional and behavioral factors that increase risk for return to substance use and poor SUD treatment outcomes. Additional work is needed to replicate these findings, determine the feasibility of widespread uptake of Transdiagnostic SUD Therapy, and examine whether the treatment effects translate to improvement in substance use outcomes.

## 1. Introduction

Substance use disorders (SUDs) are chronic, relapsing conditions with effects that persist beyond detoxification, and in some cases even years into abstinence (1). Over 20 million individuals aged 12 or older in the U.S. experience SUD in a given year (2) with a total annual cost exceeding $13 billion in hospitals alone (3). Mental and behavioral health conditions co-occur with SUDs at elevated rates (4–6), and those with dual diagnoses experience worse functional impairment and more severe substance use and psychiatric symptoms relative to those with single disorders (2). Unfortunately, only 7% of dual diagnosis patients receive concurrent treatment for their substance use and mental health disorder and of those who do seek and receive treatment for SUD, up to 75% drop out (2, 7). It is of critical importance to identify comorbid psychiatric conditions that may be concurrently targeted in SUD treatment to reduce rates of treatment dropout and relapse and to maximize desired outcomes including reduction of substance use and improvement in functional outcomes.

Anxiety (e.g., worry, nervousness, and physiological sensations) and insomnia (i.e., difficulty initiating sleep, maintaining sleep, or waking too early) represent some of the most prevalent problems among individuals with SUD. The lifetime prevalence of SUD is substantially higher among those with anxiety-related disorders [11–25% vs. 7.4%; (2, 8)], and presence of a SUD increases risk for anxiety symptoms two- to four-fold (9). Similarly, insomnia occurs in nearly all individuals with SUD through the course of use, withdrawal, and short- and long-term remission (30–91% vs. 6.4–15.0% in the general population), and lifetime history of insomnia increases risk for substance use relative to those with good sleep (4, 10–15). Substance-induced symptoms of anxiety and insomnia are also common, such as withdrawal symptoms mimicking anxiety or drug use perpetuating wakefulness (4, 16).

Anxiety and insomnia each attenuate the impact and reach of evidence-based SUD interventions. In addition to increasing probability of relapse (17–19), anxiety may also negatively impact SUD treatment seeking (20) and predict SUD treatment dropout (21, 22). Comorbid SUD-anxiety patients are most likely to receive treatment for only their substance use *or* anxiety disorder (23) and treating only SUD does not adequately reduce anxiety (19), leaving individuals vulnerable to anxiety- or stress-precipitated relapse. Further, higher levels of pre-treatment insomnia are associated with lower likelihood of SUD treatment completion (24), whereas improvement of insomnia early in SUD treatment is associated with broad improvement in overall mental health and functioning (25). For those who achieve abstinence, however, insomnia increases risk of relapse (11, 26, 27) even years into recovery (4, 28). Considering the impact of co-occurring anxiety and insomnia in SUD treatment, anxiety accounted for the relationship between alcohol use severity and insomnia (29), and 70% of patients with comorbid SUD and posttraumatic stress disorder (PTSD) experienced insomnia that did not decrease throughout course of treatment (30, 31). The available literature indicates that anxiety and insomnia both play a critical role in the efficacy (or lack thereof) of SUD treatment, and concurrently addressing both anxiety and insomnia would likely improve SUD treatment outcomes. Yet, this opportunity has not been thoroughly investigated.

Given the frequency of comorbid psychiatric symptoms (32) and high rates of relapse and dropout among those with SUDs (7, 33), transdiagnostic interventions, or principle- or process-based treatments, may effectively and efficiently address SUD *and* co-occurring emotional distress. Transdiagnostic approaches operate by targeting shared processes underlying comorbid presentations and allow for the treatment of heterogeneous clinical presentations and idiographic adaption of evidence-based intervention (34, 35). Transdiagnostic interventions have gained popularity in the last decade (36–38), yet none concurrently address anxiety *and* insomnia in the context of substance use or SUD treatment.

Many cognitive-behavioral and acceptance-based strategies have demonstrated efficacy in managing cravings, withdrawal, psychological distress, and sleep disturbances. For instance, interpersonal and emotion regulation, problem-solving, assertiveness training, behavioral activation, grounding, and relaxation strategies (e.g., deep breathing and progressive muscle relaxation) are effective in reducing anxiety, cravings, withdrawal, impulsive substance use, and improving sleep quality (39–44). Cognitive behavioral therapy for insomnia (CBT-I) is the first-line treatment for insomnia (45), however, very little empirical work has investigated CBT-I among those with SUDs. Small randomized clinical trials find that CBT-I relative to control is effective in improving subjective sleep quality and insomnia severity but not at reducing relapse (46–48). Likewise, mindfulness-based interventions reduce frequency and quantity of substance use, cravings, substance-related problems, anxiety, and experiential avoidance, and increase rates of abstinence (49–51). Despite the empirical support for each of these treatment components, research to determine the ideal selection of cognitive-behavioral and acceptance-based skills for addressing co-occurring psychiatric distress and sleep disturbances in SUD patients is needed.

In consideration of the high rates of relapse and treatment dropout among those with SUD, it is essential to target malleable factors that may otherwise increase risk for poor SUD treatment outcomes, and when treated, act as a buffer for such negative outcomes. Albeit limited, the extant literature cumulatively implicates anxiety, insomnia, and especially their co-occurrence in worse clinical and treatment outcomes among those with SUD. Additionally, group-based integrative CBT for anxiety and SUD is found to be feasible and effective, over SUD-only treatment, within a SUD-specialty IOP (52). To this end, we developed and tested a transdiagnostic, modular group intervention for reducing anxiety and insomnia in adults with SUD (Transdiagnostic SUD Therapy) based on evidence-based interventions for non-SUD populations that demonstrate effect for substance use and related outcomes. Where possible, we modified these treatment elements to be more relevant for SUD to maximize impact. We first aimed to outline the protocol for Transdiagnostic SUD Therapy, with the full protocol provided as a Supplementary File. The second aim was to pilot Transdiagnostic SUD Therapy in a single-arm trial within a real-world intensive outpatient program (IOP) for adults with SUD, most of whom were in the early stages of their recovery. We hypothesized that Transdiagnostic SUD Therapy would be associated with reductions in anxiety and insomnia severity and improvements in sleep health, which is a holistic and multidimensional pattern of sleep-wakefulness associated with promotions in wellbeing.

## 2. Materials and methods

### 2.1. Participants and procedure

Participants (*N* = 274) were referred to an IOP in the Center for Drug and Alcohol Problems (CDAP) at the Medical University of South Carolina (MUSC) after an initial triage walk-in evaluation and having completed detoxification, if necessary. MUSC is a major academic medical center in the southeastern U.S. The CDAP IOP is a 20-day program consisting of 3-h of daily group therapy and weekly individual and family therapy. See Wilkerson et al. (24) for more information about the structure of the IOP. Transdiagnostic SUD Therapy was rolled into broader IOP programming; therefore, all IOP patients had access to the Transdiagnostic SUD Therapy pilot trial if they were present in IOP treatment the day it was provided. Specifically, each Tuesday during the study period, a 90-min sleep-focused module was presented followed by a 90-min anxiety-focused module. There were no exclusion criteria for participation in the trial as all patients enrolled in the broader IOP participated in this group treatment if they were present on Tuesdays. The open trial occurred between January 2017 and April 2019 and ended due to a change in organizational structure of the IOP clinic.

A retrospective chart review was conducted to gather pertinent information. Prior to initiating IOP, participants provided demographic information and participated in interviews to assess substance use history, medical and psychiatric health history, and social history. At the beginning of each sleep module, participants were asked to complete self-report measures of insomnia and sleep health, and at the beginning of each anxiety module, participants were asked to complete a self-report measure of anxiety severity (see “Measures” below). All procedures were approved by the institution’s IRB (Pro00081480).

#### 2.1.1. Structure of Transdiagnostic SUD Therapy

Transdiagnostic SUD Therapy is comprised eight, 90-min modules including four sleep and four anxiety modules that are delivered weekly over the course of four weeks (i.e., one sleep and one anxiety module comprise a session for a total of four sessions). Corresponding sleep and anxiety modules were designed to be administered on the same day (e.g., sleep module 1 at 9 am, anxiety module 1 at 10:30 am) unless a different order is clinically indicated based on group members’ needs. The general structure of each module is consistent with that of other CBT protocols, including starting by setting the agenda. Although the content of each session differs, the agenda structure includes: (1) instructing group members to complete self-report assessments, (2) reviewing and setting sleep schedules, (3) briefly assessing use of strategies since last session, (4) presenting new material, and (5) assigning homework. Outlined below is the overarching content of each sleep and anxiety module. See Table 1 for an overview of each module and the Supplementary File for the full Transdiagnostic SUD Therapy protocol including adaptations to better address the needs of individuals with SUD.

**Table 1:** Transdiagnostic SUD Therapy overview.

Module	Session content
Sleep 1	*Sleep restriction and stimulus control*. This session follows the structure and content of a typical CBT-I introductory session. Provide information on the stages of sleep including how substances can interfere with each stage, as well as the homeostatic and circadian regulation of sleep. Explain sleep restriction and create individualized sleep schedules for each participant. Introduce stimulus control as a method for creating the context for sleep and instruct participants to only use the bed for sleeping and sex and to get out of bed if unable to fall asleep within 15 min. *Homework*: Adhere to sleep schedule and practice stimulus control.
Sleep 2	*Behavioral activation*. Sleep restriction temporarily yields increased time spent awake. Healthy use of free time and positively reinforcing substance-free activity is achieved through values-driven behavioral activation. Guide participants through a values clarification activity to demonstrate the misalignment between substance use and values and use their identified values to direct behavioral activation. Activities are recorded in a log. *Homework*: Engage in one valued activity per day.
Sleep 3	*Cognitive restructuring*. Introduce automatic thoughts and how they influence emotions and sleep. Administer a sleep beliefs quiz and discuss the group’s most common incorrect beliefs. Instruct participants how to restructure their inaccurate automatic thoughts that contribute to inaccurate beliefs about sleep (e.g., I will not sleep). *Homework*: Monitor and restructure inaccurate automatic thoughts about sleep.
Sleep 4	*Sleep hygiene*. Provide psychoeducation of the important role of sleep in recovery from substance use disorders, and how sleep can become impaired through continued use and ultimately increase risk for relapse. Present the 3P model of insomnia, and illustrate how perpetuating factors, such as substance use, can maintain and exacerbate insomnia. Introduce sleep hygiene as habits to maintain the quality and quantity of sleep (e.g., not using nicotine near bedtime, having a cool, dark, and quiet bedroom). *Homework*: Use at least one sleep hygiene principle.
Anxiety 1	*Psychoeducation and worry time*. Present psychoeducation of anxiety and worry including the etiology, psychophysiology, and presentation of anxiety disorders. Illustrate the reciprocal relationship of thoughts, emotions, and behaviors through the cognitive triangle. Generate examples from the group demonstrating how their thoughts or emotions have contributed to use. Introduce worry time and instruct participants to create a list of to-dos to each night 15 min before bed, and then set a timer starting with 5 min and increasing nightly to allow for worry and limit worry outside of this time. Instruct participants to worry for as long as they have worry, and if worrying in bed to get out and repeat the process. *Homework*: Anxiety monitoring log and daily worry time.
Anxiety 2	*Anger and regulation skills*. Discuss how not having needs met contributes to anger and increases risk for use. Identify social and personal needs in addition to physical and health-related needs. Use the cognitive triangle to demonstrate the effects and consequences of not having needs met with an emphasis on anger. Use a metaphor (e.g., boiling teapot) to demonstrate how not having needs met increases vulnerability to anger and eventually intense anger and/or aggression. Describe anger as a secondary emotion. Provide interpersonal and emotion regulation skills to prevent or intervene on anger, such as conflict resolution strategies to reduce negative consequences of anger contributing to relapse. *Homework*: Practice use of interpersonal or emotion regulation skills.
Anxiety 3	*Mindfulness and acceptance*. Demonstrate what mind*less*ness is (e.g., being on autopilot) and how it can contribute to anxiety, depression, and substance use. Provide the rationale for mindfulness and demonstrate how it can reduce anxiety and substance use. Lead participants through several mindfulness exercises (e.g., mindful observation of breath, leaves on a stream). Address any inaccurate assumptions about mindfulness (e.g., I could not get rid of my thoughts) and normalize mind wandering. Identify how mindfulness can be introduced into daily life. *Homework*: Engage in daily mindfulness.
Anxiety 4	*Relaxation strategies*. Describe the difference between calm and anxious physiology. Introduce deep breathing, progressive muscle relaxation, and grounding and explain their utility in exerting control over anxiety (*cf.* substances). Lead the group through at least one demonstration of each of the three relaxation strategies. Discuss the group’s experience after each exercise. Provide rationale for when these skills should and should not be used. *Homework*: Use relaxation strategies daily.

The information included in this table provides a high-level summary of the content included in each module. See Supplementary material for more detailed information including additional specific skills taught and more information about how to present and discuss material.

The main intervention components of the sleep modules included sleep education, sleep restriction, stimulus control, values clarification and values-consistent behavioral activation, cognitive restructuring, and sleep hygiene. The first sleep module provided education about how substances influence sleep physiology; sleep restriction therapy and stimulus control were introduced and sleep schedules for each participant were created. In sleep module 2, a value clarification activity was used to generate ideas for behavioral activation to promote healthy use of free time that is created by the sleep restriction schedule (i.e., more time spent awake). Module 3 introduced automatic thoughts about sleep and how to challenge them using cognitive restructuring. Module 4 provided education about the role of sleep and insomnia in recovery from substances and sleep hygiene was illustrated as method to maintain the quality and quantity of sleep.

Primary interventions within the anxiety modules included psychoeducation, worry time, interpersonal effectiveness skills, mindfulness and acceptance, and relaxation strategies. Anxiety module 1 used the cognitive triangle as a model for elucidating the role of anxiety and worry in substance use and risk of relapse, and worry time was introduced as a strategy to prevent worry from negatively influencing behavior and interfering with sleep initiation. In module 2, participants were provided education about how unmet needs can result in anger and subsequent relapse, and emotion regulation skills and conflict resolution strategies were introduced. Module 3 introduced mindfulness and acceptance strategies to promote present-moment awareness and to create space between emotions and impulsive substance use. The fourth anxiety module emphasized relaxation strategies, including diaphragmatic breathing, progressive muscle relaxation (PMR), and grounding techniques to cope with anxiety. Although other intervention targets (e.g., depression) and transdiagnostic processes (e.g., interoceptive exposure for anxiety sensitivity) may be appropriate to include in Transdiagnostic SUD Therapy, the needs and limitations of the IOP necessitated that we choose only two foci. We selected anxiety and sleep in part because depression was addressed in other groups and individual therapy within the IOP programming, and addressing anxiety and sleep disturbances can not only improve SUD outcomes (25, 52), but may lead to reductions in related outcomes including depression, suicidal ideation, and general emotional distress (53, 54).

#### 2.1.2. Treatment setting considerations

Relatively little research has been conducted in the implementation of evidence-based psychotherapies (EBP) in real-world clinical settings (55); therefore, little is known about whether adapting EBP in such settings maintains treatment efficacy. In some treatment contexts it may not be possible to maintain fidelity to a treatment manual. Emerging research provides support for flexible administration of manualized treatment [e.g., (56)], and the proliferation of process-based approaches calls on clinicians to deemphasize protocols and syndromes and instead utilize transdiagnostic procedures (e.g., contingency management, emotion regulation, and exposure; (34)). Rolling admission and other real-world factors inherent to an IOP necessitate that Transdiagnostic SUD Therapy be flexible and “meet patients where they are” as they present to each session. In any given week, group members may have just entered IOP and/or completed detoxification, while other members may be in their second, third, or even final week of IOP. As such, group members may or may not receive sessions in the order presented above.

### 2.2. Measures

#### 2.2.1. Generalized anxiety disorder-7

The Generalized anxiety disorder-7 (GAD-7) (57) is a 7-item self-report instrument that assesses anxiety severity over the last two weeks. Items are rated on a Likert-type scale from 0 to 3, with total scores ranging from 0 (minimal) to 21 (severe). A score of 10 indicates probable generalized anxiety disorder. The GAD-7 is positively correlated with other measures of anxiety and demonstrates excellent internal consistency among anxious (*α* = 0.92) and SUD patients [*α* = 0.93; (57, 58)] as well as in the current sample (αs = 0.91–0.95).

#### 2.2.2. Insomnia severity index

The Insomnia severity index (ISI) (59) is a 7-item self-report measure assessing perceived severity of insomnia symptoms over the last 2 weeks. Items are rated on a 5-point Likert-type scale from 0 to 4, with higher scores indicating more severe insomnia. Scores of 0–7 indicate no insomnia, 8–14 indicate subthreshold/mild insomnia, 15–21 indicate moderate insomnia, and scores above 21 indicate severe insomnia, while a score of 10 indicates clinically significant insomnia (60). The ISI demonstrates excellent internal consistency (*α* = 0.91) and a decrease in 8.4 points is indicative of moderate improvement following intervention (60). Internal consistency in the current study was good (αs = 0.86–0.89).

#### 2.2.3. Satisfaction, alertness, timing, efficiency, and duration

The Satisfaction, alertness, timing, efficiency, and duration (SATED) (61) is a 5-item self-report measure designed to assess sleep health across five dimensions—satisfaction with sleep, alertness during waking hours, appropriate timing, sleep efficiency, and duration. Each dimension is rated on a 3-point Likert-type scale from 0 (never) to 2 (usually/always) and total score ranges from 0 (“poor sleep health”) to 10 (“good sleep health”). There is no specified timeframe for rating each dimension. The SATED demonstrates acceptable internal consistency (*α* = 0.77) and is negatively correlated with measures of sleep disturbance and daytime sleepiness (62). Across the four timepoints, the SATED internal consistency was on average questionable, ranging from poor to acceptable (*α* = 0.59–0.70).

### 2.3. Data analytic plan

In total, 274 patients attended at least one Transdiagnostic SUD Therapy session, of which 111 were excluded for attending fewer than three sessions. Of note, these participants dropped out of the larger IOP at these timepoints, not specifically the Transdiagnostic SUD Therapy group. The number of patients who dropped out is large but not surprising given the high dropout rates in SUD treatment (7, 63) and outpatient treatment in particular [relative to inpatient; (64)]. The final sample consisted of 163 participants who attended three or four sessions.

As this intervention was implemented in a real-world setting as a clinical program rather than a research study, there are inconsistencies in how the data were collected on any given week. Although participants completed three or even all four sessions, they did not necessarily complete the measures for each of the attended sessions (e.g., arrived after measures were given, opted out of measures one week). As an example, a participant may have attended all four anxiety and sleep sessions but only have completed measures at two sessions or have completed the GAD-7 in all four anxiety modules but only completed the ISI and SATED in three sleep modules. We used a pre-post design to account for missing instruments, wherein the first successful administration (i.e., the first time a participant completed the measure) of the GAD-7, ISI, and SATED was designated as pre-intervention data, and the last successful administration of the three measures was designated as the post-intervention data.

Data were screened for accuracy errors and assumptions. Only individuals with complete data on all outcome measures were included in the analyses. The most common item missing on both the ISI and the SATED was the first question, which appeared on the paper-and-pencil survey as similar in font and appearance to the survey title and thus was missed/skipped over more often than the GAD-7, which more clearly delineated between the title and the survey questions. After removing participants with missing data, sample sizes in each of the final analyses were 152 for GAD-7 analyses (11 participants excluded), 132 for ISI (31 participants excluded), and 148 for SATED (15 participants excluded). Independent samples *t*-tests and Chi-square analyses examined differences in demographic variables (i.e., age, sex, race), primary substance use diagnosis, and pre-treatment anxiety, insomnia, and sleep health across patients who did (*n* = 163; 59.5%) and did not (*n* = 111; 40.5%) complete three sessions. Given low frequencies of racially/ethnically minoritized participants, race/ethnicity was dichotomized (0 = racially/ethnically minoritized, 1 = White). Descriptive statistics were used to characterize the sample, and treatment effects on anxiety, insomnia, and sleep health across the Transdiagnostic SUD sessions (i.e., pre-post) were analyzed with a series of paired samples *t*-tests. Effect sizes to determine magnitude of change on significant variables were computed using Cohen’s *d*.

## 3. Results

There were significant differences across those who completed ≥3 sessions relative to those who completed ≤2 sessions in race (*Χ^2^* [1] = 14.15, *p* < 0.001) and age (*t*[140,95] = 2.49, *p* = 0.007), such that individuals who completed ≥3 sessions were typically White and older than those who dropped out of IOP after 1 or 2 sessions. There were no differences across sex or pre-intervention anxiety, insomnia, or sleep health (*p*s > 0.05).

See Table 2 for sample demographic, substance use, and mental health information. The sample was diverse in their substance use, with alcohol use disorder (58.3%) being the primary use disorder followed by opioid use disorder (19.0%). Nearly 30% of the sample met criteria for a second use disorder, and one-in-five participants were in medication assisted treatment for substance use disorder (e.g., buprenorphine). Regarding mental health, anxiety-related disorders (including PTSD) occurred in nearly one-third of participants, while major depressive disorder was prevalent across one-fourth of the sample. At baseline, participants endorsed moderately severe anxiety (per GAD-7), clinically significant insomnia (per ISI), and moderate sleep health (per SATED), on average (Table 3).

**Table 2:** Participant pre-intervention demographics, substance use, and mental health characteristics (*N* = 163).

	% (*n*)
Age^a^	43.23 (14.57)
Sex	
Female	39.9 (65)
Male	60.1 (98)
Race	
White	95.1 (155)
Black/African American	3.1 (5)
Biracial/Other	1.2 (2)
Ethnicity	
Non-Hispanic/Latinx	100 (163)
Hispanic/Latinx	0 (0)
Primary substance use diagnosis	
Alcohol use disorder	58.3 (95)
Opioid use disorder	19.0 (31)
Cannabis use disorder	7.4 (12)
Cocaine use disorder	6.7 (11)
Amphetamine use disorder	3.7 (6)
Benzodiazepine use disorder	1.8 (3)
Secondary substance use diagnosis	
None	71.8 (117)
Alcohol use disorder	7.4 (12)
Cannabis use disorder	6.7 (11)
Cocaine use disorder	4.3 (7)
Benzodiazepine use disorder	2.5 (4)
Opioid use disorder	1.8 (3)
Amphetamine use disorder	1.2 (2)
Detoxification prior to treatment	35.0 (57)
Psychiatry appointment	55.8 (91)
Medication assisted treatment for substance use disorder^b^	21.6 (24)
Suboxone	18.0 (20)
Naltrexone	0.9 (1)
Other	2.7 (3)
None or N/A	78.4 (87)
Primary mental health diagnosis	
None	47.2 (77)
Major depressive disorder	21.5 (35)
Anxiety disorder	16.6 (27)
Attention-deficit hyperactivity disorder	4.3 (7)
Bipolar I disorder	4.3 (7)
Posttraumatic stress disorder	2.5 (4)
Secondary mental health diagnosis	
None	72.4 (118)
Anxiety disorder	12.3 (20)
Major depressive disorder	3.1 (5)
Posttraumatic stress disorder	3.1 (5)
Attention-deficit hyperactivity disorder	1.8 (3)
Bipolar I disorder	1.2 (2)
Bipolar II disorder	1.2 (2)
Schizophrenia	0.6 (1)

^a^M(SD). ^b^*n* = 111.

**Table 3:** Descriptive statistics and paired samples *t*-tests regarding intervention effects on anxiety, insomnia, and sleep health from pre- to post-intervention.

	*n*	Pre-intervention		Post-intervention		*t* (df)	*p*	95% CI	Cohen’s *d*
		Mean	*SD*	Mean	*SD*				
Anxiety	152	11.19	6.16	7.00	5.95	9.29 (151)	< 0.001	3.32–5.11	0.753
Insomnia	132	10.42	6.00	6.17	5.38	9.17 (131)	< 0.001	3.33–5.16	0.798
Sleep health	148	6.49	2.47	7.78	2.21	−6.42 (147)	< 0.001	−1.69 to −0.893	−0.528

Anxiety = CI = confidence interval; GAD-7; insomnia = ISI; sleep health = SATED. Sleep health is scored from 0 to 10, with higher values indicating greater sleep health (i.e., the expected direction following intervention). Therefore, the *t*-test statistic and Cohen’s d values would be negative if the intervention led to improvements in sleep health as hypothesized.

**Figure 1:**
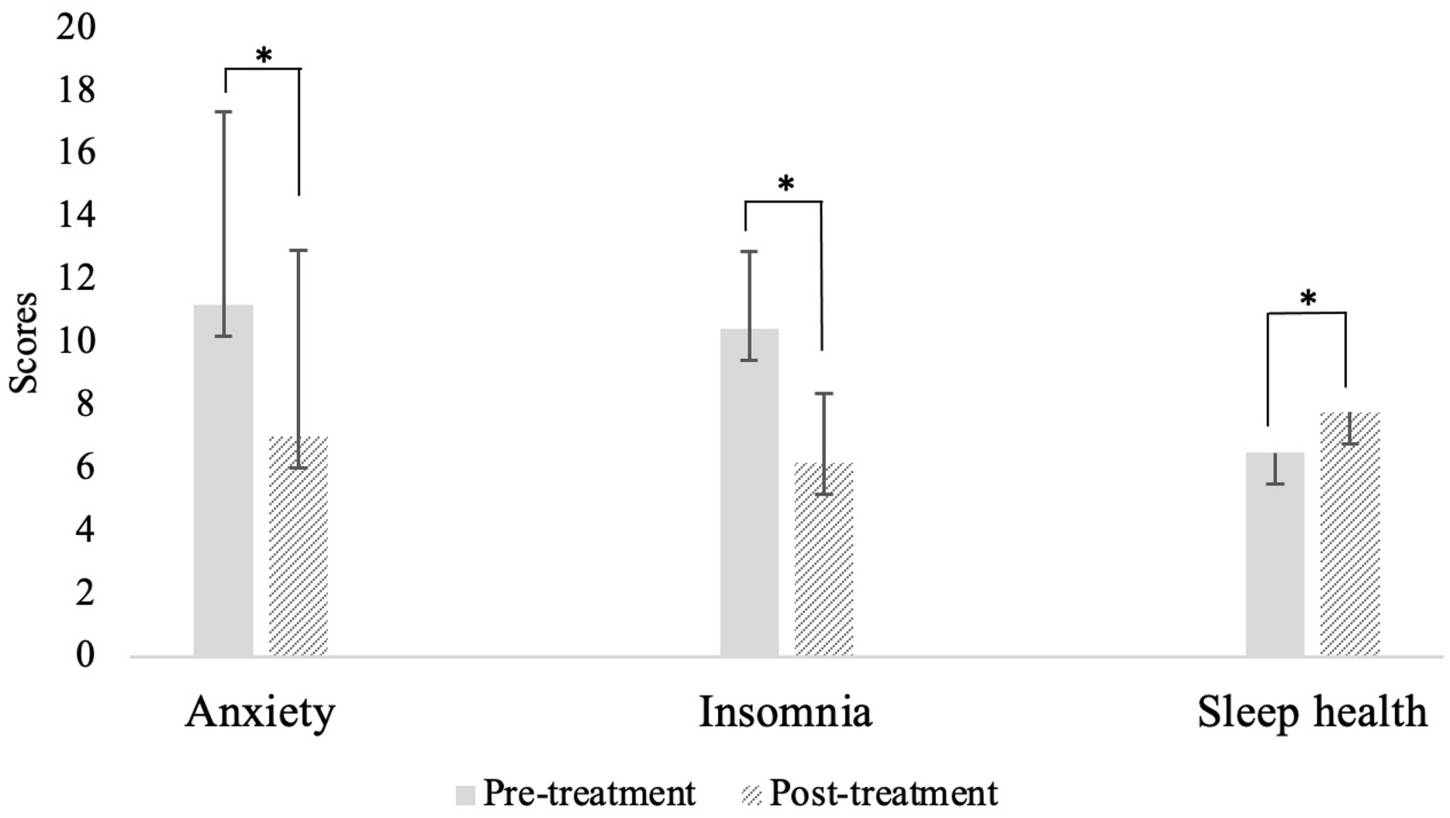
Means and standard error of anxiety, insomnia, and sleep health from pre-to post-intervention. *p < 0.001.

See Table 3 for paired samples *t*-test statistics. Anxiety, insomnia, and sleep health all significantly improved from pre- to post-intervention (Figure 1).^1^ Specifically, anxiety and insomnia both reduced to below clinical significance and sleep health improved throughout the course of Transdiagnostic SUD Therapy. Notably, these findings demonstrated medium to large effects (*d*s > 0.5). To gather a more fine-grained picture of sleep health, we examined change in individual SATED dimension means across each of the four timepoints. All sleep health dimensions increased over each of the four sessions except Timing, which increased from session 1 to session 2 and then slightly decreased and plateaued at sessions 3 and 4. All dimensions of sleep health, on average, increased from the first administration to the last administration, with Satisfaction increasing the most (0.58 points) followed by Efficiency (0.53), Duration (0.47), Alertness (0.16), and Timing (0.14).

## 4. Discussion

The aims of this manuscript were to describe the protocol and evaluate preliminary support for a transdiagnostic group intervention for anxiety and insomnia among intensive outpatients receiving treatment for SUD. Patients experienced significant reductions in anxiety and insomnia severity and improvement in sleep health throughout the 4-week group intervention. Notably, these findings remained across sex and primary SUD, indicating Transdiagnostic SUD Therapy is effective for both biological males and females and across different types of SUD. Prior to implementation of Transdiagnostic SUD Therapy in this IOP, standard IOP patients experienced clinically significant insomnia symptoms at week 4 of IOP, and reductions in anxiety were 70% less than what was observed in the current study (25). Though the present study did not have a control group, our findings suggest that the implementation of Transdiagnostic SUD Therapy in this IOP may in part contribute to clinical improvement of IOP patients. Importantly, as our sample had various alcohol/substance use and mental health diagnoses, Transdiagnostic SUD Therapy may have the potential to be effective regardless of type of substance(s) used or mental health condition, highlighting its impact and utility as a transdiagnostic intervention. In other words, individuals with SUDs do not necessarily need to have anxiety or sleep disorders to benefit from this intervention, likely given the high prevalence of anxiety and insomnia symptoms throughout the cycle of substance use and recovery. Given anxiety and insomnia occur in withdrawal through early recovery from substances (2, 11), this intervention can supplement traditional SUD treatment by concurrently addressing modifiable factors associated with treatment dropout and relapse (27, 52).

In addition to their prevalence in SUDs, insomnia and anxiety co-occur at heightened rates. Insomnia is the most common sleep disturbance among those with anxiety disorders (up to 50% comorbidity), and individuals with insomnia are at increased risk for anxiety up to 17-fold (65, 66). Insomnia and anxiety reciprocally influence one another, such that lack of sleep can exacerbate distress and diminish one’s ability to cope, while anxiety prolongs sleep onset through negative repetitive thinking and/or physiological arousal. Despite this frequent co-occurrence and bidirectional relationship, insomnia requires targeted intervention, such as CBT-I, to evidence improvements. Indeed, CBT-I is superior to anxiety-focused treatment in improving insomnia symptoms among those with comorbid anxiety disorders and shows moderate to large effects in reducing comorbid anxiety symptoms (67, 68). However, up to 30% of individuals with comorbid anxiety do not experience insomnia-related benefit from CBT-I (69), suggesting that additional intervention components may be needed to mitigate co-occurring anxiety. Cognitive behavioral and acceptance-based interventions are equally effective in the treatment of anxiety disorders (50, 70, 71) and in combination with CBT-I, may be the ideal synergy of strategies to simultaneously improve insomnia and anxiety symptoms. Our findings reflect this sentiment, with integrated elements of CBT-I, CBT for anxiety, and acceptance- and mindfulness-based strategies associated with robust reductions in anxiety and insomnia among SUD patients. However, additional work is needed to examine within-individual differences in anxiety and insomnia outcomes of Transdiagnostic SUD Therapy as different treatment trajectories across individuals may uncover the need for additional, optional Transdiagnostic SUD Therapy modules and/or adaptations to diverse presentations or different clinical settings. Our results speak to this need, in that that racially/ethnically minoritized and younger individuals were less likely to complete ≥3 weeks of IOP than their White and older counterparts. Additionally, whether these treatment components influence substance use outcomes remains to be seen and is the focus of ongoing data collection and analyses.

A major benefit of transdiagnostic interventions is simultaneously addressing overlapping symptoms of comorbid presentations with a single approach (72, 73). Concurrently addressing comorbidities results in better outcomes than single-disorder protocols or treating comorbidities in a sequential manner. For example, Concurrent Treatment of PTSD and Substance Use Disorders Using Prolonged Exposure (COPE) contributes to reductions in PTSD symptoms and SUD severity (74, 75) and yields better SUD outcomes than SUD-only treatment (76). Yet, the relations of sleep disturbance and anxiety among individuals with SUD are complex and little research to date has investigated all three conditions simultaneously, and none have in an intervention context. Experimental studies indicate that anxiety-related factors (e.g., rumination, anxiety sensitivity) may be potential mechanisms influencing the relationship between sleep disturbances and substance use (29, 77, 78), but this work is limited and does not examine the reciprocal role of insomnia on anxiety and substance use. Taken together, this scant literature suggests that transdiagnostic treatment approaches for addressing SUD can be effective and are sorely needed, and that Transdiagnostic SUD Therapy may be a promising approach. Randomized controlled trials are needed to examine the impact of Transdiagnostic SUD Therapy on sleep, anxiety, and substance use outcomes relative to treatment as usual or control.

In addition to improving insomnia symptoms, Transdiagnostic SUD Therapy also appears to contribute to improvements in sleep health. On average, satisfaction, efficiency, and duration of sleep evidenced the greatest improvements across the intervention period. While insomnia consists of difficulty with initiating and maintaining sleep (1), sleep health represents a multidimensional perspective of sleep-wakefulness encompassing physical, mental, and neuro-behavioral wellbeing (61). Rather than focusing on disordered or deficient sleep, sleep health emphasizes positive and holistic sleep characteristics by identifying how *well* an individual is sleeping (61). As sleep health is negatively correlated with insomnia severity (79), it is not surprising that Transdiagnostic SUD Therapy evidenced improvements in sleep health among our sample. Because sleep health is multifaceted and tied to both sleep-related and overall health, it is possible that the combination of CBT-I and anxiety-focused strategies included in Transdiagnostic SUD Therapy contributed to improvement of sleep health. Empirical work is needed to understand what intervention components influence sleep health. Preliminary evidence implicates increases in sleep onset latency as a salient predictor of declining sleep health (80), suggesting that treatments aimed to enhance sleep health should focus on reducing sleep onset latency.

Preliminary results suggest that Transdiagnostic SUD Therapy may be feasible and effective when administered in a real-world IOP for SUD. Dropout, defined in the current study as attending fewer than three of four total sessions, was approximately 40%, which is similar to or lower than rates found throughout the published literature. A recent meta-analysis indicated that the average dropout rate for in-person psychosocial SUD treatment is 30%, but there is wide variability depending on several demographic and substance use characteristics and can reach as high as 75% (7). Treatment length may be an advantage of 4-session Transdiagnostic SUD Therapy, as fewer sessions are associated with lower rates of dropout (7). Transdiagnostic SUD Therapy is designed to be rolled into existing SUD treatment programming, in this case IOP but other modalities (e.g., individual therapy) and settings (e.g., outpatient, residential) may be appropriate. Integrating Transdiagnostic SUD Therapy along with patients’ attempts to reduce or cease use of substances is recommended given the prevalence of anxiety and insomnia symptoms during withdrawal and early abstinence. Implementing Transdiagnostic SUD Therapy simultaneously with reductions in substance use can provide skills needed to cope with cravings or withdrawal symptoms that may occur at this time, as well as act as a prophylactic method of mitigating triggers for substance use associated with anxiety and/or sleep disturbance that commonly arise during withdrawal and early recovery from substances. Rigorous clinical trials will be needed to examine whether Transdiagnostic SUD Therapy is feasible, acceptable, and effective in alternative settings and in addressing withdrawal and cravings in early SUD treatment. An appropriate next step may be to seek key patient and provider input on the structure (e.g., number of sessions) and intervention elements (e.g., treatment targets) of Transdiagnostic SUD Therapy to determine the optimal composition. Additional trials are needed to determine feasibility of implementation on a larger scale across multiple clinics, as well as designing internally valid, controlled trials to systematically examine additional treatment outcomes and associated transdiagnostic moderators.

### 4.1. Limitations and future directions

Limitations of Transdiagnostic SUD Therapy and the preliminary data presented inform next steps in research and clinical work. Potentially most critical is the homogenous sample, in which nearly all group members identified as White. The effects of this treatment should be replicated within a more racially/ethnically diverse sample to understand the intervention’s efficacy among minoritized individuals, particularly in the context of the finding that ethnically/racially minoritized individuals were less likely to attend at three or more sessions. Cultural adaptations of Transdiagnostic SUD Therapy are likely warranted to account for racial disparities in SUD treatment, and subsequent clinical trials should prioritize recruiting a more diverse sample. In this vein, additional work is needed to understand the impact of other social determinants of health on risk for substance use and the success of Transdiagnostic SUD Therapy, including factors such as socioeconomic status, transportation access, and health literacy. Additionally, a strength of this investigation is the ecologically valid implementation of Transdiagnostic SUD Therapy in a real-world SUD treatment setting. However, clinical trials are needed to investigate whether the cognitive behavioral and acceptance-based strategies selected for Transdiagnostic SUD Therapy are the most effective combination for this population, and the development of additional optional modules for specific populations (e.g., racial/ethnic minorities), motivations for substance use (e.g., coping, provoke/prevent sleep), and other psychiatric symptoms (e.g., depression, posttraumatic stress) may be warranted. Systematic trials may also provide insight into treatment gains provided by Transdiagnostic SUD Therapy above those offered by standard IOP programming. Given the transdiagnostic nature of Transdiagnostic SUD Therapy, inclusion of additional outcome measures to assess global effects (e.g., depression, PTSD symptoms), transdiagnostic mechanisms (e.g., anxiety sensitivity, emotion regulation), and substance use outcomes (e.g., cravings) is warranted to better understand mechanisms of change and scope of intervention effect. Such information can help to refine the intervention to maximize its impact on a wide array of substance use and co-occurring emotional disorders (e.g., need for inclusion of behavioral activation for depression). Further, although over half of our sample were meeting with a psychiatrist, we do not know what psychotropic medications (other than medication assisted treatment) participants were prescribed, if any. Psychotropic medications, such as antidepressants or tranquilizers, could contribute to declines in anxiety and insomnia symptoms; thus, future research should record this information and control for medication effects. In a similar vein, we are underpowered to conclude how medications prescribed for substance use disorders may have influenced the anxiety and sleep findings. Given these medications can reduce anxiety and exacerbate sleep disturbance (81, 82), future research is needed to understand how they may interact with Transdiagnostic SUD Therapy. Finally, 40.5% of the patients who began the Transdiagnostic SUD Therapy (and broader IOP) only completed one or two Transdiagnostic SUD Therapy sessions. These patients were typically racial/ethnic minorities and younger. Future randomized controlled investigations of Transdiagnostic SUD Therapy should seek to examine whether these differences stand and whether other sociodemographic and clinical factors may be contributing to premature dropout. Preliminary results presented herein highlight that Transdiagnostic SUD Therapy is effective across sexes, yet additional work is needed to evaluate whether this intervention is equally effective for sexual and gender diverse individuals. Additionally, researchers should aim to identify how many sessions are needed to experience clinically and statistically significant improvements in anxiety and insomnia symptoms.

### 4.2. Conclusion

Transdiagnostic SUD Therapy may reduce anxiety and insomnia symptoms and improve overall sleep health across sex and various SUDs among adults enrolled in IOP for SUD. These findings meaningfully contribute to the SUD treatment literature by providing preliminary evidence for a brief transdiagnostic intervention that may concurrently address two non-substance-related factors that contribute to relapse and poor SUD treatment outcomes. Further, given that increases in symptoms of anxiety and insomnia are common during active use, withdrawal, early recovery, and even sustained abstinence (12, 83, 84), this protocol may be appropriate for any individual with a history of substance use or SUD, regardless of having an anxiety or insomnia disorder. Our findings suggest that Transdiagnostic SUD Therapy is a promising intervention for integration into real-world SUD treatment settings. Though, replication is needed in community-based settings and randomized controlled trials are warranted to establish its efficacy in not only reducing anxiety and insomnia symptoms but improving substance-related outcomes such as treatment retention, harm reduction/abstinence, and sustained recovery.

## Data availability statement

The raw data supporting the conclusions of this article will be made available by the authors, without undue reservation.

## Ethics statement

The studies involving human participants were reviewed and approved by Medical University of South Carolina Institutional Review Board. Written informed consent was not provided because data were gathered *via* a retrospective chart review, for which IRB approval was granted.

## Author contributions

SW and AW contributed to writing on the first draft of the manuscript. MM ran intervention groups and was responsible for data collection. JP and KH assisted with running intervention groups. MM and AW contributed to the design of the intervention. SW, TM, and AW acquired data from EMR and ran analyses. All authors contributed to major editing and approved the submission of the manuscript.

## Funding

This research was supported by the National Institute on Drug Abuse of the National Institutes of Health (2K12 DA031794) awarded to AW.

## Conflict of interest

The authors declare that the research was conducted in the absence of any commercial or financial relationships that could be construed as a potential conflict of interest.

## Publisher’s note

All claims expressed in this article are solely those of the authors and do not necessarily represent those of their affiliated organizations, or those of the publisher, the editors and the reviewers. Any product that may be evaluated in this article, or claim that may be made by its manufacturer, is not guaranteed or endorsed by the publisher.

## Footnotes

^1^We used a series of ANCOVAs to examine whether there were differences in post-intervention anxiety, insomnia, and sleep health across sex and primary SUD diagnosis controlling for pre-intervention symptoms. All models were nonsignificant (*p*s > 0.07).
