# Supplementary material for: A Transdiagnostic group therapy for sleep and anxiety among adults with substance use disorders: Protocol and pilot investigation
Source: Front Psychiatry. 2023 Mar 29;14:1160001. doi: 10.3389/fpsyt.2023.1160001 (PMC10090550; doi:10.3389/fpsyt.2023.1160001)
Supplement: Supplementary file 1 [file Data_Sheet_1.docx]

Supplementary File 1: Detailed protocol for Transdiagnostic SUD Therapy.

**Sleep Modules**

Each sleep session begins by reviewing the principles of sleep restriction and stimulus control to reinforce their importance, promote adherence, and emphasize the role of sleep in recovery from drugs and alcohol. For returning group members, review the previous week’s sleep schedule and adjust individuals’ sleep schedules as needed using CBT-I guidelines for increasing time in bed (TIB; see Taylor et al., 2019). For those having trouble with these components (which in our experience encompasses many patients), solicit problem solving feedback from the group. For new group members, create a sleep restriction schedule by using data provided by a sleep diary or by conducting a quick assessment for those who have not completed a sleep diary.

**Sleep Session 1.** The purpose of this session is to provide psychoeducation about sleep and introduce sleep restriction and stimulus control as a method of consolidating sleep and standardizing sleep and wake time. This session generally follows a typical CBT-I introductory session protocol (e.g., Taylor et al., 2019). Introduce the stages of sleep and their benefits, including body repair and memory consolidation, and how substances can negatively influence sleep. Stage 3 sleep (otherwise known as deep sleep or slow wave sleep) promotes rest and repair of the body and brain, while REM sleep promotes memory consolidation. Without enough sleep or with fragmented sleep, we cannot gain the amount of stage 3 and REM sleep that is needed, and thus lack the benefits of sleep. Importantly, substances negatively influence sleep cycling. Alcohol causes an increase in stage 2 sleep and reduction in stage 3 sleep, while tranquilizers (e.g., Ambien) cause an individual to skip REM sleep and enter straight into stage 3, preventing the mind repair benefit of REM sleep.

Next, describe the basic physiology of sleep, including the two process models of sleep and wake (i.e., the homeostatic [process S] and circadian [process C] regulation of sleep), to convey what comprises good sleep before introducing how sleep can become disrupted. Regarding the latter, not accumulating enough sleep debt (process S) prior to bedtime (i.e., not being tired enough to fall asleep) and being exposed to light at night (process C), such as through a phone or television, promote wakefulness and contribute to insomnia through creating fragmented sleep, extended sleep onset latency, and preventing acquisition of enough deep sleep.

Sleep restriction therapy is introduced as a method for making sleep more efficient through reducing the amount of TIB by matching TIB to the amount of sleep an individual needs. We use hunger as parallel example to sleep – we can train our body to become hungry at certain times just as we can train our bodies to become tired at certain times. Individualized sleep schedules are created for each group member based on their amount of sleep needed. Explain to group members that sleep restriction is temporary; once they begin to sleep more efficiently in this window, their TIB will be expanded to allow for more sleep. Each group member should be given a sleep dairy to compete over the next week. Lastly, stimulus control is introduced as a method of setting the environment for sleep, and the guidelines for stimulus control are presented: 1) only get into bed when sleepy and intending to fall sleep, 2) only use the bed/bedroom for sleep and sex, 3) leave the bedroom when awake for more than 15 minutes, 4) only return to the bedroom when sleepy enough to fall asleep, and 5) adhere to your sleep and wake time set through sleep restriction sleep schedule. Assign each group member a sleep restriction schedule for the following week. Troubleshoot and problem solve as needed and review any barriers that may prevent group members for adhering to their new sleep schedules and stimulus control guidelines.

**Sleep Session 2.** In addition to no longer spending time obtaining, using, or recovering from substances, sleep consolidation provides more free time throughout the day and into the evening. Therefore, behavioral activation is introduced to promote healthy use of free time, identify positively reinforcing substance-free activity, increase energy, and prevent sleeping as a response to avoidance of triggers, cravings, or unwanted emotions (e.g., boredom, anxiety). Taking turns, elicit from each group member their ideas for daily behavioral activation activities for the following week. We used and adapted the “Valued Directions Worksheet” from *The Mindfulness & Acceptance Workbook for Anxiety* (Forsyth & Eifert, 2007) to facilitate group members’ identification of value directed behaviors, although any values clarification worksheet may be used.

Substance use in the context of SUD is based in avoidance and aversive control, often precluding engagement in valued behavior (Stotts & Northrup, 2015). Field and colleagues (2020) posit that individuals with SUDs may overcome addiction by shifting their reinforcing preferences from substance use to substance-free activity through engaging in values-based decision making. Through use of the “Valued Directions Worksheet,” we aimed to highlight the misalignment between substance use and one’s values with the goal of facilitating identification and engagement in values-directed behavior. To do this, we first asked participants to list the 10 domains of satisfaction (e.g., work/career, parenting, spirituality) in order of level of current engagement, then in order of priority. Typically, those with SUDs will experience a misalignment in how important a value is and how frequently they are engaging in that value. Highlight this discrepancy and describe how this misalignment contributes to anxiety and depression, which may then trigger use. Then, generate ideas for behavioral activation using each group member’s most important valued domain. Once identified, group members should write their daily activities in a log and/or put it in a calendar to increase adherence.

**Sleep Session 3.** In this session introduce automatic thoughts, their effect on emotions and sleep including their role in exacerbating insomnia, and how to replace unhelpful automatic thoughts with more accurate or balanced thoughts (i.e., cognitive restructuring). Elicit examples of automatic thoughts about sleep from the group members. Common automatic thoughts among patients with insomnia include: I won’t sleep, I won’t be able to function tomorrow, I’ll go crazy or get sick, and I’ll be awake the whole night. We adapted the Dysfunctional Beliefs and Attitudes about Sleep (DBAS), a 16-item self-report measure assessing the degree to which certain beliefs about sleep are believed, into a true/false “quiz” and administered it to group members (all correct answers are “false”). Ask group members to tally how many items they selected “true” and how many items they selected “false.” On a whiteboard, write the numbers 1 through 16 to correspond with each DBAS item, and write the total number of group members who put “true” for each item. Inform members all answers are “false,” and discuss the top 3-4 items (as time allows) that the group collectively answered incorrectly, identifying why the statements are false and eliciting alternate, more accurate thoughts. Use the “Common Dysfunctional Thoughts and Realistic Alternatives” handout to facilitate this discussion. For homework, instruct group members to monitor their inaccurate automatic thoughts about sleep and attempt to restructure them using the handouts as a guide.

**Sleep Session 4.** Additional psychoeducation of the importance of sleep in recovery and how sleep becomes impaired, as well as sleep hygiene principles are discussed. The importance of addressing sleep disturbance in early recovery from substances is identified. Other than the role of sleep in the repair and recovery of the brain and body, illustrate that using affects the quality of sleep, and sleep can similarly negatively affect early recovery from substances. Specifically, poor sleep may serve as a trigger or an avoidance strategy, cause anxiety or depression, and impair control over cravings, all of which may result in increased risk of relapse. We then present the 3P model of insomnia (Spielman et al., 1987), which describes the predisposing (i.e., genetic or environmental influences that increase risk for insomnia), precipitating (i.e., a stressor or event contributing to the onset of insomnia), and perpetuating (i.e., behavioral coping factors that maintain insomnia) factors of insomnia. Perpetuating factors are targeted through treatment, such as maintaining a regular sleep schedule and only using the bed for sleep and sex (i.e., sleep restriction therapy and stimulus control). Lastly, sleep hygiene involves habits that help to maintain the quality and quantity of sleep and include the following: not drinking caffeine after noon; not drinking alcohol near bedtime (which is consistent with the overarching treatment goal of harm reduction or abstinence); not using nicotine near bedtime; exercising in the morning or 3-6 hours before bedtime; having a cool, dark, and quiet bedroom environment; eating a light snack at bedtime; and avoiding drinking 8 ounces or more of fluids within 2-3 hours of bedtime.

***Anxiety Modules***

**Anxiety Session 1.** This session introduces psychoeducation of anxiety and worry, including the etiology, psychophysiology, and presentation of anxiety disorders. Begin with a discussion of how anxiety can be both “good” (e.g., provides information, motivation, and protection from harm) and “bad” (e.g., negative impact on sleep, impair choices, limit behavior), eliciting specific examples from group members. Identify a middle ground (i.e., anxiety is neither good nor bad) through linking anxiety to recovery (e.g., anxiety can help alert us to risk for relapse).

The cognitive triangle is used to illustrate how thoughts, emotions, and behaviors influence each other, and how worrisome thoughts can contribute to self-fulfilling prophecies (i.e., confirmation of an anxious thought through avoidant behavior). Beginning with a general example (e.g., you see a friend while out shopping and the friend ignores you), illustrate how thoughts influence behavior and emotion. Elicit from the group examples of thoughts that might arise in this situation (e.g., friend is mad at me, friend didn’t see me), identifying how each thought leads to a different emotion (e.g., anxiety, neutral) and behavior (e.g., ignore friend, approach friend), and how behavior may influence others’ behavior (e.g., friend walks away, friend approaches you). Emphasize how our behavior influences the behavior of other people and can result in a self-fulfilling prophecy of our original worrisome thought (e.g., because I didn’t approach my friend and instead walked away now my friend is upset with me, confirming the original thought ‘my friend is mad at me’). Ask group members to generate examples of self-fulfilling prophecies from their recent past and discuss as a group. Then, apply the self-fulfilling prophecy to substance use, emphasizing that how one interprets a situation influences cognitions about the situation, behaviors in the situation (including avoidance and use), and other people’s behaviors, ultimately confirming the original thought and contributing to substance use.

Lastly, introduce worry time, wherein group members were instructed to create a list of to-dos each night and set a timer for at least 5 minutes to journal their worries, with the intention of limiting worry outside of this time. Although they were instructed to set a timer for 5 minutes, the group members were encouraged to journal worries for up to 30 minutes. In wrap up, elicit from the group how they can use the information learned about the self-fulfilling prophecy and worry time in their recovery. For homework, provide a worry and anxiety log to track the three components of emotions and have group members identify a consistent time each day for worry time.

**Anxiety Session 2.** In this session, we identify how having needs met (or not) yields differential emotional experiences, and introduce emotion regulation and interpersonal effectiveness skills to effectively navigate getting needs met and coping with emotions. Begin by soliciting a list of needs from each group member. Often, physical and health needs are identified (e.g., food, shelter), but other needs such as social and personal needs are neglected. Discuss the difference between needs and wants, emphasizing that things like love and companionship are important needs that when not met can yield unwanted psychological distress. Provide a handout illustrating the comprehensive nature of human needs and describe how distinct emotional experiences are associated with having these needs met or stymied. When reviewing different emotions, use the cognitive triangle as a model to identify the various components of each distinct emotion, highlighting how they are similar or different from one another (e.g., anxiety and excitement share similar physiological sensations). Through this process, identify cognitions that can prevent one’s needs from being met, such as “because of my using and that I’ve hurt people I love, I don’t deserve my needs being met.”

Next, discussion how anger is often the emotional response to not having needs met. Effective regulation of anger and/or assertiveness can be problematic for individuals with SUDs or in early recovery from SUDs and may yield risk for relapse. Elicit from the group examples of when their needs were not met, resulting in anger and subsequent substance use. Using an analogy such as a boiling teapot, describe how not having needs met increases vulnerability to anger (e.g., simmering water) and continuing to not meet needs contributes to aggression and more intense anger (e.g., water starts to boil out of control). Additionally, describe how anger is a secondary emotion, in that it is typically occurs following a more painful or “weaker” emotion. For example, anger is often perceived by those with SUDs as a “safer” emotion relative to emotions like feeling hurt, guilty, or rejected, which may be perceived as showing weakness.

Last, discuss how anger can contribute to aggression and identify negative consequences of aggression (e.g., pushing people away, continuing to prevent needs being met, relapse). Describe to the group how the emotion of anger itself is not problematic, but that it is verbal and/or physical behavior that can result in negative consequences. Assertive, aggressive, and passive responses are identified as learned behaviors, and therefore are not unchangeable. The conflict resolution model is introduced, and group members practice each step (i.e., identifying the problem, identifying the feelings, identifying the specific impact, deciding whether to resolve the conflict, and addressing and resolving the conflict) and are given feedback by the rest of the group.

**Anxiety Session 3.** The purpose of this session is to introduce mindfulness and acceptance strategies for anxiety and worry. Begin by asking participants for instances in which they have been ‘on autopilot,’ such as when driving and not remembering the drive once they arrive at the destination. Using this example, illustrate the concept of schemas, wherein the brain creates shortcuts to save time and allow us to reserve mental energy. However, these shortcuts can lead to inaccurate appraisals, such as “I need to drink to be fun” or “I can’t have friends because I hurt people,” which contribute to not only using but increased anxiety and depression. Additionally, when on autopilot, group members may not observe triggers for using, which can contribute to relapse.

Mindfulness is introduced as a strategy to maintain attention in the present, allowing for a greater understanding of what is occurring in the here and now, rather than getting caught up in the past (rumination) or future (worry). Emphasize that the goal of mindfulness is not to achieve a state of relaxation or to “clear the brain” or “turn the brain off.” Mindfulness is defined to the group as paying attention, purposefully, without judgment, in the moment, with the goal of building a different relationship with one’s experiences (i.e., rather than avoiding, being present with one’s experiences; Kabat-Zinn, 1994, 2015). Explain to group members that mindfulness is a skill that, when practiced consistently, can reduce anxiety. Through mindfulness, acceptance can be achieved, which is defined as intentionally making contact with one’s emotions without trying to hold onto (such as with happiness or excitement) or push away (such as with anger, sadness, or anxiety) such experiences. The distinction between acceptance and approval or acknowledgement is discussed. Each group member identifies and discusses what acceptance might look like for them.

Lead the group through mindfulness and acceptance exercises, such as mindful observation of breathing and more active practices like “leaves on a stream.” Elicit reactions from the practice from each group member. For group members who may not have liked the practice, explore why and correct any inaccurate assumptions (e.g., I wasn’t able to get rid of a thought, I don’t feel any more relaxed). For many group members, this is the first time they have practiced mindfulness; normalize that mindfulness is a difficult skill to develop and wandering thoughts is expected. Review ways in which mindfulness may be practiced in daily life (e.g., meditation or yoga, on a walk, eating, other daily activities) and ask each group member to practice mindfulness over the next week.

**Anxiety Session 4.** Relaxation strategies for coping with anxiety are introduced, including PMR, deep breathing (e.g., 4-count breathing, box breathing, diaphragmatic breathing), and grounding. These relaxation strategies are contrasted from simply taking a breath, which patients often report they are told to do by their friends and family when they are stressed. The physiology of relaxation versus anxiety is described, including their differential effects on adrenaline, heart rate, breathing, tension, digestion, perspiration, blood pressure, and blood flow. Describe to group members how practicing relaxation strategies can help them to exert control over their anxiety in a more effective and sustainable way than drugs and alcohol can. Emphasize to group members that these skills should be practiced frequently and consistently to receive the full benefit.

Lead the group through three exercises to illustrate how these skills work to reduce tension or stress. First, use a PMR script (approximately 10-15 minutes) to illustrate the benefit of relaxing the muscles, particularly around the head and upper torso where tension is often held. Next, conduct a brief deep breathing practice, instructing group members that the goal is to mimic the rate of breathing during sleep, which is 5 cycles of deep breathing over the course of 60 seconds (i.e., 5-7 breaths in one minute). Before and after each exercise, elicit from each group member what their stress level is (e.g., from 0, no stress, to 10, extreme stress), and illustrate how spending just a few minutes practicing relaxation can decrease their stress level in the moment and without the help of drugs or alcohol.

Finally, introduce grounding as a means to anchor oneself in the present when experiencing intense emotions and/or urges or cravings to use (or other destructive behavior) so as to avoid impulsive action such as using. Describe the three methods of grounding oneself, including physical, mental, and self-soothing. When describing the utility of grounding, provide a rationale of what grounding is and what it is not (i.e., avoidance). In other words, emphasize that grounding is a short-term strategy to get through a tough moment, akin to distress tolerance. Next, engage the group in practicing or instructing them on how they can practice each of the three methods of grounding, providing several examples of each. Before beginning grounding practice, instruct group members identify something they have been worried about or a particularly challenging thought. Physical grounding can be practiced by holding ice, dumping one’s face in a bowl of ice water, taking a very cold or hot shower, or using worry stones. Mental grounding can be illustrated in group by asking group members to start at a random number and repeatedly subtracting 17, or by asking them to write down as many types of berries as they can think of in a 30 second period. Lastly, self-soothing ideas can be generated by the group, including affirmations or positive self-talk (e.g., “this too shall pass”) and self-care activity incorporating physical sensations (e.g., taking a bubble bath, lighting a candle, eating a favorite snack, going for a walk). After practicing grounding in group, ask the group members how often they thought about their original worry or thought. Use this practice to illustrate how grounding can be used as a distraction from extremely intense emotions or risky situations such as when they are experiencing a craving to use. Instruct group members to practice relaxation skills regularly and grounding techniques as needed over the next week.

**A Comment on Sleep Diaries**

In traditional CBT-I protocols, daily sleep diaries are an important element of treatment that document baseline sleep severity, identify factors that may affect nightly variations in sleep, and monitor treatment progress. Ideally, patients would begin completing sleep diaries at least one week prior to initiating CBT-I and be provided instructions to fill out sleep diaries as soon after waking as possible to capture sleep data most accurately, with an explanation of the rationale for completing sleep diaries provided in the first session.

In the real-world clinical setting in which Transdiagnostic SUD Therapy is designed to be administered in, it may not be realistic to expect that all group members will complete and return sleep diaries each week. To ensure all patients are progressing in their sleep restriction schedule and adhering to stimulus control guidelines regardless of sleep diary completion, clinicians must be able to perform quick assessments of all group members’ sleep (in our experience, group members rarely returned sleep diaries). Still, clinicians should emphasize the importance of completing sleep diaries and preemptively problem solve the issue of forgetting to complete them by identifying a time or location to fill in the diary or by suggesting setting reminders or using visual cues to promote completion of the sleep diary each day. It is also important to describe to group members how to fill out the sleep diary (or informally track sleep); for instance, differentiating time in bed from the time they intend to try to fall asleep. Note that the group members are not expected to watch the clock to provide accurate data, but rather, emphasize that they should do their best to guess or estimate their responses.
